# Spatial contagion during the first wave of the COVID‐19 pandemic: Some lessons from the case of Madrid, Spain

**DOI:** 10.1111/rsp3.12522

**Published:** 2022-03-16

**Authors:** María Hierro, Adolfo Maza

**Affiliations:** ^1^ Department of Economics University of Cantabria Spain

**Keywords:** COVID‐19, inter‐municipal mobility, Madrid, spatial contagion, spatial dependence

## Abstract

This paper analyses the magnitude of the spatial transmission of COVID‐19 through the municipalities of the region of Madrid during the first pandemic wave using a spatial contagion index. The study also provides additional insights into the main factors contributing to the spread of the virus in both time and space by estimating a novel conditional spatial contagion index. Our results reveal high values of spatial contagion before and during the national lockdown enacted on 15 March 2020, becoming medium/low since then. Furthermore, the study confirms the leading role of inter‐municipal mobility and population density in spatial contagion.

## 1 INTRODUCTION

The study of the coronavirus disease 2019 (COVID‐19) pandemic, from different perspectives and/or approaches, has been one of the most prolific topics in regional science since its outbreak in late 2019/early 2020. Within this topic, a considerable amount of research has been conducted on the identification of risk factors for COVID‐19 infection on the basis of clinical and ecological studies. Although the results are mixed, many of these studies confirm the disproportionate impact of COVID‐19 in the most deprived communities, especially in certain demographic groups such as the elderly and certain ethnicities, and in territories with low income levels (Plümper & Neumayer, 2020; Ribeiro et al., 2020). Recent literature also reveals the significant role of urban population share, tourism, CO_2_ emissions, high average temperatures and human mobility in explaining COVID‐19 outbreaks (Bijari et al., 2021; Hâncean et al., 2020). Another point of interest emerging from this branch of the literature is related to the important distinction between population and population density. While some studies conclude that infections are significantly correlated not with density but with population levels (e.g., Boterman, 2020), other empirical studies reveal a positive relationship between population density of the different areas and COVID‐19 incidence (Buja et al., 2020; Mogi & Spijker, 2021). As Desmet and Wacziarg (2020) indicate, the way population density is measured strongly affects the results obtained.

In any event, as far as the study of the pandemic is concerned, it is meaningful to point out that something differentiates the health crisis unleashed by COVID‐19 from previous crises, such as those caused by an ordinary economic shock: it does not distinguish between territories. We are talking about a virus with a vast capacity for contagion between people, which ends up spreading contagion between territories. In other words, the geographical mobility of people (whether for work, leisure or other reasons) facilitates the transmission of the virus between territories, with the intensity of the contagion process depending, as we shall see and in line with some previously mentioned papers, not only on the degree of mobility of its population but also, to a greater or lesser degree, on the characteristics of the territory. These factors make the COVID‐19 pandemic a complex phenomenon, to which no territory is immune, and in which spatial interactions undoubtedly play an important role.

In this framework, the use of spatial econometric techniques provides a very powerful tool to address the main spatio‐temporal patterns of COVID‐19 spread. Moreover, such techniques can be used to oversee the design and implementation of measures aimed at curbing the spread of the disease and, more importantly, to assess the importance of emerging hotspots of COVID‐19 that should be closely monitored to prevent further expansion of the virus through and/or between territories. Accordingly, spatial econometric tools have been widely employed in recent years when dealing with this topic. We can cite the studies by Maza and Hierro (2021), Paez et al. (2021), Arenas et al. (2020), Cordes and Castro (2020), Dehghan and Shahnazi (2020), Ghosh and Cartone (2020), Guliyev (2020), Kang et al. (2020), Krisztin et al. (2020) and Orea and Álvarez (2020), among many others. The most notable and predictable conclusion of all these studies is that space, that is, the geographic location of the units of analysis, clearly matters.

This paper seeks to contribute to the growing body of literature addressing the dimension and main drivers of the spatial spread of COVID‐19, but from a different angle: quantifying spatial contagion and analysing, on the basis of that measure, the importance of various factors in explaining it. To do so, it uses municipal‐level data for the region of Madrid during the first pandemic wave (6 March to 21 June 2020), a critical period that has not been sufficiently covered by the extant literature. As the increasing concern about the emergence of new pandemics is becoming a prominent theme in both public and academic discourses (Telenti et al., 2021), this study may be fruitful for the management of forthcoming pandemics, especially in the initial phases to quickly contain the spread of a virus in the absence of a vaccine, or even, as is happening at the time of writing, in situations like the current one in which the efficacy of a vaccine in stopping the propagation of a disease is limited.

Specifically, in the first part of the paper, we resort to the concept of spatial contagion defined by Hierro et al. (2013) on the grounds of a spatial Markov chain approach, adapted here to the case of an epidemiological disease. This concept is particularly attractive as it goes far beyond a simple measure of community transmission. It refers to contagion between territories over time. More precisely, it considers territories that may be more likely exposed to a spatial/geographical contagion process, that is, those that are surrounded by others with a significant contagion capacity. It is, therefore, a concept that informs us on how spatial interactions contribute to the spread of the virus from high‐ to low‐incidence areas throughout a region. As a further contribution, in the second part of the paper, we extend this approach by defining a new measure of conditional spatial contagion that allows us to evaluate the role played by some potential drivers in the virus propagation, such as inter‐municipal mobility (see below), population (number of inhabitants), population density (inhabitants per squared kilometre), income (gross disposable income) and immigration (stock of foreign population). To our knowledge, this is the first study to assess the scale and key drivers of COVID‐19 spatial contagion.

Another relevant contribution and a key strength of the study lies in the use of novel mobility data (recorded from mobile phones) provided by the Spanish National Statistical Institute (INE). These data are rich enough to allow us to address in detail the influence of inter‐municipal mobility on spatial contagion processes. The main value of these data is that they enable us to assess the effect of all types of mobility on spatial contagion processes, including commuting, shopping trips and leisure. Thus, we join other researchers who have used mobile positioning data to analyse the impact of mobility on the COVID‐19 propagation in countries such as Italy and Brazil, among many others (Peixoto et al., 2020; Pepe et al., 2020).

The remainder of this paper is as follows. Section 2 introduces the case study and the dataset. The methodology is explained in Section 3, while the results are presented in Section 4. The paper concludes in Section 5 with a summary of the main lessons learned in the light of the evidence collected in this paper.

## 2 CASE STUDY AND DATASET

### 2.1 Case study

The study area comprises the region of Madrid (Spain) at the municipal level. Specifically, we deal with the different phases of the first pandemic wave, with special emphasis on the role played by the mobility of people between municipalities. The case study is particularly relevant since Madrid is deemed the ‘epicentre’ of the pandemic in Spain, with a balance of 69,732 confirmed cases and 8,369 deaths between 6 March and 21 June 2020, accounting for over 26% and 29% of the total figures in Spain, respectively. Nor can we disregard that, despite its widely recognized health services, Madrid's health care system shouldered the highest pressure across the country.

It was the end of February 2020 when the circulation of the virus became evident in the region of Madrid. Faced with an escalation of cases, the president of the region introduced, in early March, some social distancing measures, such as the suspension of classroom teaching in schools and universities, and the prohibition of family visits to nursing homes. A few days later, on 15 March, the Spanish government decreed a national lockdown to battle the coronavirus, marking the beginning of the so‐called state of alarm. All non‐essential shops were forced to close, and mobility was restricted to the purchase of essentials and urgent medical assistance. One week later, on 23 March, the collapse of Madrid's hospitals and intensive care units (ICUs) urged the regional government to erect (in less than 48 h) a kind of field hospital at the *Institución Ferial de Madrid* (IFEMA) conference centre, designed to host 5,500 hospital beds and 500 intensive care beds.

**FIGURE 1:**
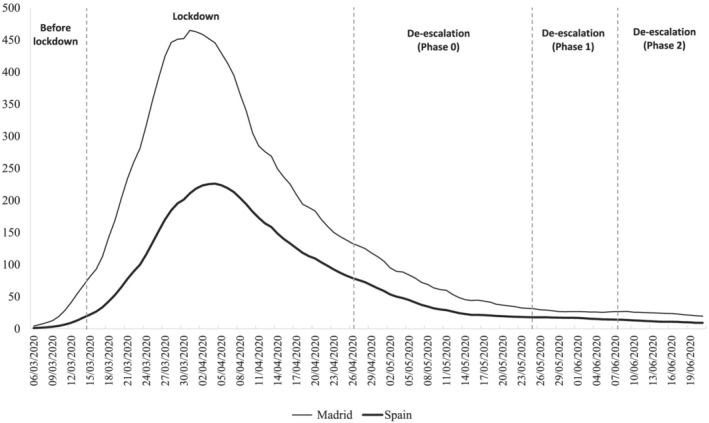
Cumulative incidence of COVID‐19: The region of Madrid and Spain *Note*: Dates of the de‐escalation period refer to Madrid

Although the national lockdown was initially envisaged for 2 weeks, the tragic death toll forced the Spanish government to extend it for an additional 4 weeks. As Figure 1 shows, the huge gap between the 14‐day cumulative incidence (defined as the number of cumulative cases reported in the last 14 days per 100,000 inhabitants) in Madrid and Spain 1 became disturbingly large at the height of the health crisis, with the incidence in Madrid lying very close to the critical threshold of 500 (almost double the national average). It is clear, therefore, that Madrid acted as the first major focus of the virus in Spain. After 6 weeks of strict lockdown marred by dramatic and brutal numbers of fatalities in both the region and the whole country, the Spanish government designed a coordinated plan to pave the way towards a kind of normalcy in which clampdowns were progressively loosened in an orderly fashion. In this manner, the so‐called de‐escalation phase began, conducted in three‐phased stages. Thereafter, the cumulative incidence indicator dropped significantly in Madrid. At the beginning of the de‐escalation phase, for instance, it had fallen to just 125. The end of this phase, on 21 June, marked the end of the state of alarm.

### 2.2 Variables, data sources and sample period

Our main variable of analysis is the 14‐day cumulative incidence of confirmed COVID‐19 cases (defined above), herein denoted by *CI*. This variable is calculated using data on daily counts of confirmed cases at the municipal level collected from the Open Data Portal of the Community of Madrid. Specifically, we gather information for 178 municipalities, as well as for the 21 districts that compound the metropolitan area of the municipality of Madrid. Here it is necessary to clarify that the region of Madrid is made up of 179 municipalities, although the one with the same name as the region is clearly, in both extension and population, an outlier. For this reason, we collect information for the remaining 178 municipalities, as well as for the 21 districts that compound the municipality of Madrid.

To unveil the main drivers of spatial contagion, we also collected data on inter‐municipal mobility, population, population density, gross disposable income and immigrant population. For other potential drivers mentioned in the Introduction, such as CO_2_ emissions, temperature and so on, either there are no data at the municipal level or the differences between municipalities are not significant. Data on inter‐municipal mobility consist of mobility registered over the previous 14 days, in an analogous fashion as our measure of cumulative incidence. This aspect is extremely relevant because it is well recognized that the peaks of virus prevalence during the lockdown were the natural consequence of the high mobility that occurred in the previous weeks. To estimate this variable, we exploit data on inter‐municipal mobility, as well as inter‐district mobility (for some relatively big municipalities) and inter‐census areas mobility (for the biggest one, the municipality of Madrid) coming from ‘Studies on mobility based on mobile phone’ published by the INE. This information is based on data recorded on mobile phones, so it includes all types of mobility (commuting, shopping trips and leisure). For more details on the exploitation of the data source, see the Appendix. The remaining variables come from the Statistical Institute of the Community of Madrid and the Madrid City Council website.

The sample period starts on 6 March 2020, as this is the first day before the declaration of the national lockdown with more than one municipality with a 14‐day cumulative incidence of confirmed COVID‐19 cases per 100,000 inhabitants over 25 [threshold from which risk of contagion exists according to the European Centre for Disease Prevention and Control (ECDC)], and extends to 21 June 2020, the last day of the state of alarm. This implies a sample of 108 days across 199 spatial units (namely 178 municipalities plus the 21 districts that make up the municipality of Madrid). According to the different stages of the first wave mentioned above, we divide the entire period into five sub‐periods: before lockdown, lockdown, de‐escalation (phase 0), de‐escalation (phase 1) and de‐escalation (phase 2).

## 3 METHODS

### 3.1 Spatial dependence

To formally test for the existence of spatial interactions in the virus propagation, we resort to the well‐known Moran's *I* statistic, which can also be represented graphically in what is known as the Moran's scatterplot. To do so, we first pre‐define the spatial connectivity structure of the sample through the so‐called spatial weights matrix W, with generic elements $w_{\mathrm{ij}}$ called spatial weights. In our case, $w_{\mathrm{ij}}$ is defined as the inverse of the distance between centroids of municipalities i and j, so that interactions between them decay inversely with distance. 2 As is convention, we use standardized spatial weights, so that each element of matrix W is divided by its row sum. The Moran's *I* statistic at a given time t reads as follows:

(1) $$I\left(t\right)=\frac{n}{\sum_{i=1}^{n}\sum_{j=1}^{n}w_{\mathrm{ij}}}\frac{\sum_{i=1}^{n}\sum_{j=1}^{n}w_{\mathrm{ij}}\left[{\mathrm{CI}}_{i}\left(t\right)-\mathrm{CI}\left(t\right)\right]\left[{\mathrm{CI}}_{j}\left(t\right)-\mathrm{CI}\left(t\right)\right]}{\sum_{i=1}^{n}{\left[{\mathrm{CI}}_{i}\left(t\right)-\mathrm{CI}\left(t\right)\right]}^{2}}\;\text{for}\;i\neq j,$$

where ${\mathrm{CI}}_{i}$ ( ${\mathrm{CI}}_{j}$) is the cumulative incidence at municipality i ( j), CI refers to the sample mean and n is the total number of municipalities.

### 3.2 Spatial contagion index

As mentioned before, we adapt the notion of spatial contagion proposed by Hierro et al. (2013). Specifically, here we tackle situations in which municipalities surrounded by others with relatively high COVID‐19 incidence (those with higher‐than‐average cumulative incidence values) move towards higher cumulative incidence levels.

Consequently, to construct our measure of spatial contagion, we first need to define the spatial lag for each municipality i. It is computed as the weighted‐average cumulative incidence in neighbouring municipalities, that is, ${\mathrm{CI}}_{i}^{*}\left(t\right)=\sum_{j}w_{\mathrm{ij}}{\mathrm{CI}}_{j}\left(t\right)$, so that we can identify municipalities surrounded by others with a higher‐than‐average cumulative incidence. The rationale behind this notion is to integrate space into virus transmission by deriving a weighted average of the neighbouring observations.

Then, let us suppose a modified spatial Markov chain approach 3 in which municipalities surrounded by others of high incidence at t (namely municipalities with a spatial lag variable above the regional average at t, denoted hereafter by k) 4 are grouped into a finite number of exhaustive and mutually exclusive states $S=\left\{s_{i\left(j\right)}:\;i,j=1,2,\ldots ,r\right\}$ according to their cumulative incidence at times t and t+z, so that $X_{k}\left(t\right)$ and $X\left(t+z\right)$ represent the cumulative incidence state/interval occupied by them at time t and t+z, respectively. Then, we can define the spatial transition probability between a cumulative incidence state $s_{i}$ to another $s_{j}$ between t and t+z as:

(2) $$p_{\left.s_{i}s_{j}\right|k}\left(t,t+z\right)=\text{prob}\left[\left.X\left(t+z\right)=s_{j}\right|X_{k}\left(t\right)=s_{i}\right].$$

In other words, if we consider the cumulative incidence state $s_{i}$, then $p_{\left.s_{i}s_{j}\right|k}\left(t,t+z\right)$ will give us the probability of municipalities within that state, surrounded by above‐average incidence municipalities at time t, of moving towards the state $s_{j}$. All the spatial transition probabilities are gathered in the so‐called spatial transition matrix $P_{k}\left(t,t+z\right)$.

Accordingly, the spatial contagion index for the period $\left(t,t+z\right)$, denoted as $C_{k}$, can be defined as follows:

(3) $$C_{k}\left(t,t+z\right)=\sum_{s_{i}}\sum_{s_{j}>s_{i}}p_{\left.s_{i}\right|k}\left(t\right)p_{\left.s_{i}s_{j}\right|k}\left(t,t+z\right)d_{\left.s_{i}s_{j}\right|k}\left(t\right),$$

where $p_{\left.s_{i}\right|k}\left(t\right)$ represents the initial proportion of municipalities surrounded at t by high‐incidence areas and grouped in $s_{i}$'s cumulative incidence state 5; $\;p_{\left.s_{i}s_{j}\right|k}\left(t,t+z\right)$ are the spatial transition probabilities defining upward mobility ( $s_{j}>s_{i}$); and, finally, $d_{\left.s_{i}s_{j}\right|k}\left(t\right)$, the generic element of a distance matrix $D_{k}\left(t\right)$, is a distance measure between states $s_{i}$ and $s_{j}$ that captures how far a cumulative incidence state is from another at time t, and defined as the difference in average cumulative incidence of the states considered. To get a normalized index, distances $d_{\left.s_{i}s_{j}\right|k}\left(t\right)$ are divided by the largest value in the $s_{i}$'s row of $D_{k}\left(t\right)$. By construction, the spatial contagion index ranges between zero and one: the closer its value is to one, the higher the level of spatial contagion.

From Equation 3, we can deduce for each state $s_{i}$ what could be called a state‐by‐state spatial contagion index as:

(4) $$C_{k}^{s_{i}}\left(t,t+z\right)=\sum_{s_{j}>s_{i}}p_{\left.s_{i}s_{j}\right|k}\left(t,t+z\right)\;d_{\left.s_{i}s_{j}\right|k}\left(t\right)$$

which captures the contribution of each state to the spatial contagion process.

### 3.3 Conditional spatial contagion index

As for the main factors that can expose municipalities surrounded by areas with relatively high COVID‐19 incidence to higher levels of cumulative incidence over time, in this paper we propose a conditional/weighting scheme based on the estimation of spatial transitional probabilities conditional on a factor f that can potentially influence spatial contagion, that is, $p_{\left.s_{i},s_{j}\right|k}^{f}\left(t,t+z\right)$. The rationale is as follows: rather than simply considering the number of municipalities moving from one state to another in the estimation of the transition probabilities, we now use additional information coming from the conditioning factor. As a result, we will have two spatial contagion indexes: one unconditional (the previous $C_{k})$ and another conditional ( $C_{\left.k\right|f})$.

**FIGURE 2:**
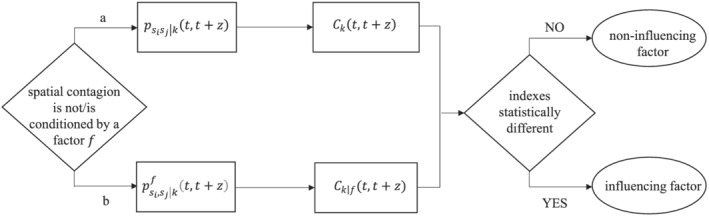
Main steps in the identification of factors that may influence spatial contagion processes

Therefore, the influence of a factor on spatial contagion would be reflected in the differences between the two indexes. If a factor had no importance on municipal movements across the cumulative incidence intervals, values for the conditional and unconditional transition probabilities should be roughly the same, and consequently, other things being equal, values for the unconditional and conditional spatial contagion indexes should be equal as well. Otherwise, if the conditional index were significantly lower (higher) than the unconditional, one would have to conclude that spatial contagion and the conditioning factor f are related, the relationship being negative (positive). By way of synthesis, Figure 2 displays a flow chart describing the main steps of our approach.

## 4 RESULTS

### 4.1 Spatial distribution of the virus: Spatial dependence

**FIGURE 3:**
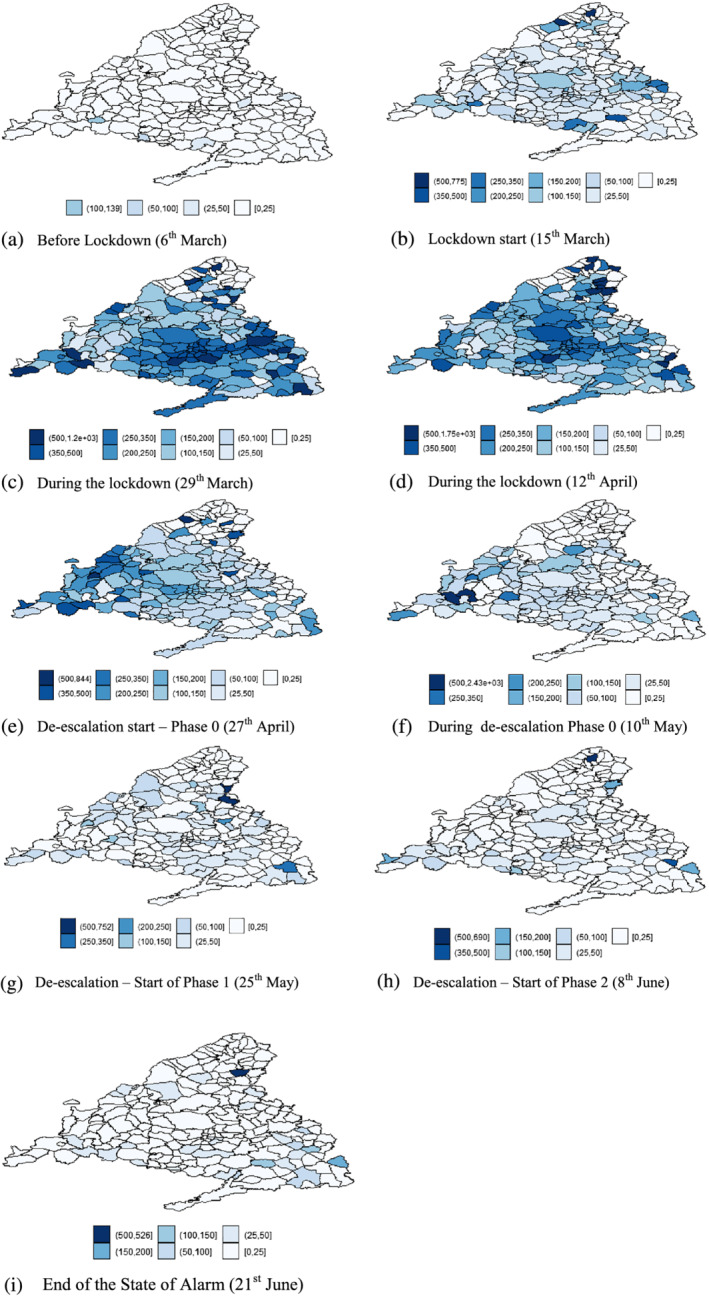
Cumulative incidence of COVID‐19 in Madrid by municipality *Note*: As indicated in the legend, different shades correspond to different 14‐day cumulative incidence intervals of confirmed COVID‐19 cases per 100,000 inhabitants

To get a first glimpse of how the spread of the virus evolved in Madrid, Figure 3 shows the geographical distribution of the cumulative incidence by municipality over the sub‐periods considered. The sequential comparison of the maps perfectly illustrates the uncontrolled propagation of the pandemic in Madrid (with progressively darker maps) until well into phase 0 of de‐escalation, with the spread of the disease slowing down thereafter (with increasingly lighter maps). Without being exhaustive, three key conclusions merit special attention. First, as Figure 3b shows, on the day the lockdown officially began, the virus was already widespread throughout most of the region, with 54% of territories with a cumulative incidence higher than 25 cases per 100,000 inhabitants. In fact, some hotspots of a high incidence of COVID‐19 had already emerged, although still very scattered. These findings inevitably raise the question: was the establishment of the lockdown too late? In our opinion, the figures seem to support an affirmative answer, at least as far as Madrid, the focus of the pandemic in Spain from day 1, is concerned. Second, the slowdown and subsequent stabilization of the transmission of the virus were not achieved until well into phase 1 of the de‐escalation (late May). In our view, this seems to reveal the merit of extending phase 0 to control the spread of the virus and thus prevent it from flaring up again. Third, Figure 3 seems to convey the idea that the cumulative incidence of COVID‐19 is not randomly distributed among municipalities. To be more precise, it seems to be closely related to that in their neighbouring municipalities, especially in some sub‐periods. The clearest case of spatial clustering is illustrated, in the centre of the maps, by the districts that make up the municipality of Madrid – recall that we divided it by size and population – and its adjacent municipalities during the lockdown phase.

Our last conclusion is, however, speculative at best, and should be tested statistically. Hence, we resort here to the Moran's *I* statistic. Table 1 presents this statistic for some representative dates throughout the period of analysis. As can be seen, its sign is positive and statistically significant only from well into the lockdown phase until before the end of the de‐escalation phase 0, so the existence of spatial association was restricted to part of our sample period. Our study provides support to the expected link between these results and the spatial contagion processes we will address later.

**TABLE 1:** Cumulative incidence of COVID‐19 in Madrid: Spatial autocorrelation test across municipalities

Phase	Date	Moran's I (*)
Before lockdown	6 March	0.000 (0.282)
Lockdown	15 March	−0.004 (0.491)
	29 March	0.049 (0.000)
	12 April	0.022 (0.000)
De‐escalation (phase 0)	27 April	0.059 (0.000)
	10 May	0.018 (0.000)
De‐escalation (phase 1)	25 May	0.002 (0.136)
De‐escalation (phase 2)	8 June	−0.012 (0.847)
End of nationwide state of alarm	21 June	−0.008 (0.692)

*Note*: (*) Figures in brackets indicate the corresponding p− value.

**FIGURE 4:**
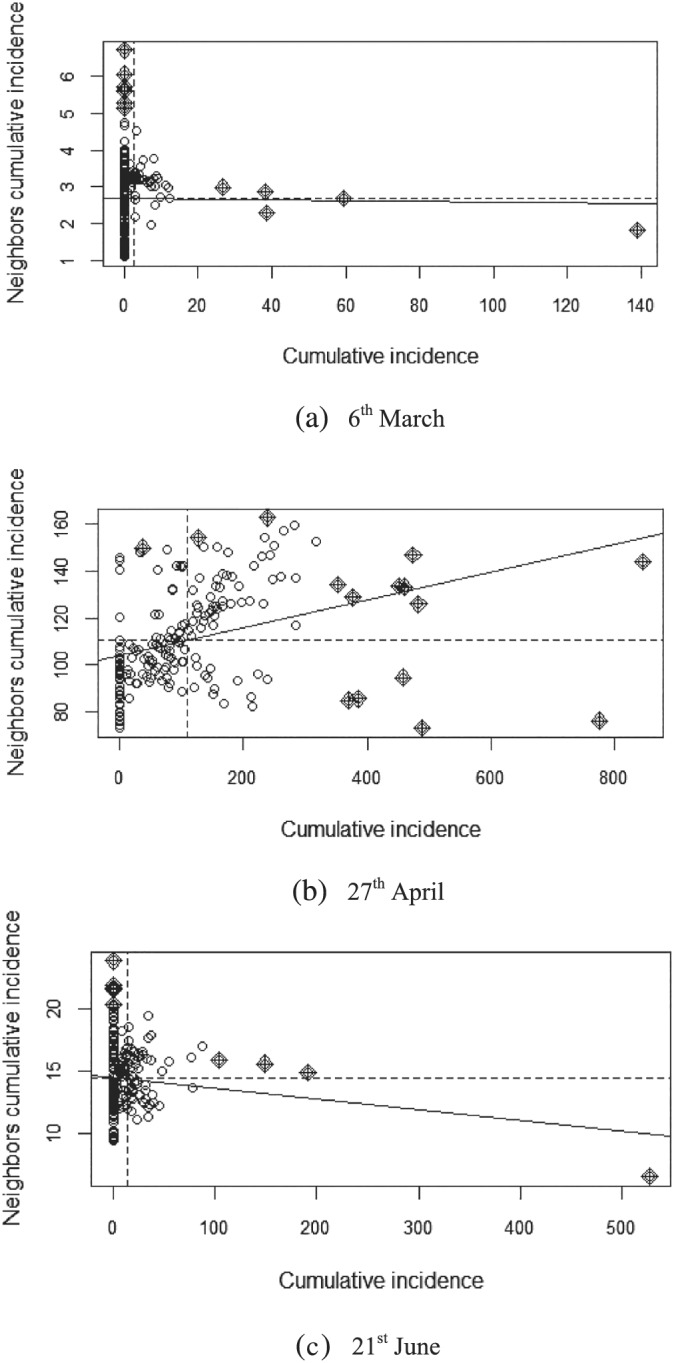
Cumulative incidence of COVID‐19 in Madrid: Moran's scatterplot *Note*: Rhomboid‐shaped black dots represent influential points (‘inliers’)

Next, we turn to the Moran's scatterplot shown in Figure 4, as it is a useful graphical tool that allows us to visualize more intuitively the spatial association patterns revealed by the Moran's *I* statistic (Anselin, 1995). It charts the spatial lag on the vertical axis and the value at each location on the horizontal axis, both at time t. The four quadrants in the plot correspond to different types of spatial association. For example, the first quadrant (upper left) represents a negative association of municipalities with low cumulative incidence surrounded by others with high cumulative incidence, while the upper‐right quadrant represents a positive association of high values. This graphical tool, however, provides only a static picture of the issue at hand, so we will include some dynamics by looking at different dates. For the sake of simplicity, Figure 4 depicts Moran's scatterplot only for the initial date, one intermediate date (end of the lockdown) and final (end of the state of alarm) date of our sample period.

By comparing the position of the points in the sequential Moran's scatterplots, we can observe important changes from the upper‐left to the upper‐right quadrant, suggesting that many spatial contagion processes took place during the first COVID‐19 wave, especially in its first half. In any case, caution is required because a cursory glance at Figure 4 is not enough to draw solid conclusions. It can be deemed as a preliminary overview that provides, nevertheless, some intuition on the issue at hand. Thus, the next aim is, on the one hand, to give a measure of the intensity of the contagion and, on the other, to unveil some of its driving factors.

### 4.2 Intensity of contagion: Spatial contagion index

For this purpose, we first present in Table 2 the spatial transition matrices, $P_{k}\left(t,t+7\right)$, the relative size of the states, $p_{\left.s_{i}\right|k}\left(t\right)$, the state‐by‐state spatial contagion indexes, $C_{k}^{s_{i}}\left(t,t+7\right)$, and, finally, the spatial contagion index, $C_{k}\left(t,t+7\right)$, for the five sub‐periods into which we divided our period, as well as for the whole one. Before commenting on the results, it is worth making some preliminary considerations on the definition of the states and the length of the transition period, since both must be pre‐determined by the researcher. As for the states, in our case intervals of *CI*, we use the following nine intervals: lack of risk [0,25], very low risk (25,50], low risk (50,100], medium‐low risk (100,150], medium risk (150,200], medium‐high risk (200,250], high risk (250,350], very high risk (350,500] and, finally, extreme risk (500, +∞). Finally, regarding the length of the transition period, Hierro et al. (2013) recommend choosing neither a very short nor a very long length for transitions. This is because the former would imply very limited mobility, while a too long one would lead to a significant loss of observations. Under the above premise and considering that the analysis of the spatial spread of a virus requires a minimum time frame (of at least, we think, 1 week), we opt for a 7‐day transition period ( z=7).

**TABLE 2:** Cumulative incidence of COVID‐19 in Madrid: Spatial transition matrices and spatial contagion indexes

a) Before lockdown ( k=3.396)									
$s_{i}/s_{j}$	[0,25]	(25,50]	(50,100]	(100,150]	(150,200]	(200,250]	(250,350]	(350,500]	(500, +∞)
[0,25]	0.470	0.286	0.190	0.030	0.000	0.012	0.006	0.006	0.000
(25,50]	0.000	0.000	0.500	0.500	0.000	0.000	0.000	0.000	0.000
(50,100]	0.000	0.000	0.333	0.000	0.000	0.000	**0.667**	0.000	0.000
(100,150]	0.000	0.000	0.000	0.000	0.000	0.000	0.000	0.000	0.000
(150,200]	0.000	0.000	0.000	0.000	0.000	0.000	0.000	0.000	0.000
(200,250]	0.000	0.000	0.000	0.000	0.000	0.000	0.000	0.000	0.000
(250,350]	0.000	0.000	0.000	0.000	0.000	0.000	0.000	0.000	0.000
(350,500]	0.000	0.000	0.000	0.000	0.000	0.000	0.000	0.000	0.000
(500, +∞)	0.000	0.000	0.000	0.000	0.000	0.000	0.000	0.000	0.000
$p_{\left.s_{i}\right|k}\left(t\right)$	**0.971**	0.012	**0.017**	0.000	0.000	0.000	0.000	0.000	0.000
$C_{k}^{s_{i}}\left(t,t+7\right)$	**0.064**	**0.139**	**0.357**	0.000	0.000	0.000	0.000	0.000	0.000
$C_{k}\left(t,t+7\right)$	**0.0703**								

b) Lockdown ( k=185.511)									
$s_{i}/s_{j}$	[0,25]	(25,50]	(50,100]	(100,150]	(150,200]	(200,250]	(250,350]	(350,500]	(500, +∞)
[0,25]	0.704	0.030	0.051	0.081	0.034	0.022	0.020	**0.024**	**0.032**
(25,50]	0.218	0.141	0.269	0.167	0.192	0.000	0.013	0.000	0.000
(50,100]	0.108	0.066	0.354	0.271	0.157	0.036	0.006	0.000	**0.003**
(100,150]	0.086	0.055	0.202	0.290	0.211	0.083	0.061	**0.011**	0.000
(150,200]	0.020	0.023	0.143	0.233	0.273	0.182	0.082	**0.032**	**0.012**
(200,250]	0.011	0.002	0.101	0.216	0.315	0.178	0.142	**0.029**	**0.005**
(250,350]	0.035	0.006	0.027	0.096	0.170	0.227	0.254	**0.154**	**0.030**
(350,500]	0.031	0.000	0.015	0.006	0.050	0.163	0.287	0.356	**0.092**
(500, +∞)	0.127	0.000	0.007	0.033	0.040	0.051	0.105	0.185	0.451
$p_{\left.s_{i}\right|k}\left(t\right)$	0.126	0.020	0.092	0.138	0.153	0.113	0.167	0.122	0.070
$C_{k}^{s_{i}}\left(t,t+7\right)$	0.086	0.072	0.050	0.050	0.054	0.032	0.065	0.085	0.000
$C_{k}\left(t,t+7\right)$	**0.0570**								

c) De‐escalation: Phase 0 ( k=102.198)									
$s_{i}/s_{j}$	[0,25]	(25,50]	(50,100]	(100,150]	(150,200]	(200,250]	(250,350]	(350,500]	(500, +∞)
[0,25]	0.872	0.079	0.031	0.009	0.001	0.000	0.002	0.001	0.005
(25,50]	0.485	0.482	0.033	0.000	0.000	0.000	0.000	0.000	0.000
(50,100]	0.280	0.431	0.240	0.037	0.000	0.000	0.012	0.000	0.000
(100,150]	0.111	0.102	0.574	0.130	0.000	0.056	0.009	0.009	0.009
(150,200]	0.156	0.031	0.547	0.063	0.094	0.016	0.063	0.000	0.031
(200,250]	0.125	0.083	0.167	0.500	0.042	0.000	0.000	0.042	0.042
(250,350]	0.160	0.120	0.240	0.280	0.040	0.000	0.120	0.040	0.000
(350,500]	0.120	0.000	0.200	0.120	0.080	0.040	0.160	0.240	0.040
(500, +∞)	0.455	0.000	0.182	0.000	0.000	0.000	0.091	0.000	0.273
$p_{\left.s_{i}\right|k}\left(t\right)$	0.495	0.202	0.149	0.065	0.039	0.014	0.015	0.015	0.007
$C_{k}^{s_{i}}\left(t,t+7\right)$	0.012	0.001	0.005	0.021	**0.042**	**0.053**	0.007	0.040	0.000
$C_{k}\left(t,t+7\right)$	**0.0115**								

d) De‐escalation: Phase 1 ( k=25.671)									
$s_{i}/s_{j}$	[0,25]	(25,50]	(50,100]	(100,150]	(150,200]	(200,250]	(250,350]	(350,500]	(500, +∞)
[0,25]	0.877	0.077	0.027	0.003	0.005	0.003	0.000	0.000	0.008
(25,50]	0.523	0.432	0.045	0.000	0.000	0.000	0.000	0.000	0.000
(50,100]	0.404	0.213	0.383	0.000	0.000	0.000	0.000	0.000	0.000
(100,150]	0.467	0.267	0.000	0.267	0.000	0.000	0.000	0.000	0.000
(150,200]	0.000	0.000	0.000	0.000	0.800	0.000	0.000	0.000	0.200
(200,250]	0.000	0.000	0.000	0.333	0.000	0.667	0.000	0.000	0.000
(250,350]	0.000	0.000	0.000	0.000	0.000	0.000	0.000	0.000	0.000
(350,500]	0.000	0.000	0.000	0.000	0.000	0.000	0.000	0.000	0.000
(500, +∞)	0.000	0.000	0.500	0.000	0.333	0.000	0.000	0.000	0.167
$p_{\left.s_{i}\right|k}\left(t\right)$	0.662	0.201	0.085	0.027	0.009	0.005	0.000	0.000	0.011
$C_{k}^{s_{i}}\left(t,t+7\right)$	0.016	0.002	0.000	0.000	**0.200**	0.000	0.000	0.000	0.000
$C_{k}\left(t,t+7\right)$	**0.0130**								

e) De‐escalation: Phase 2 ( k=20.345)									
$s_{i}/s_{j}$	[0,25]	(25,50]	(50,100]	(100,150]	(150,200]	(200,250]	(250,350]	(350,500]	(500, +∞)
[0,25]	0.918	0.051	0.027	0.004	0.000	0.000	0.000	0.000	0.000
(25,50]	0.426	0.515	0.029	0.029	0.000	0.000	0.000	0.000	0.000
(50,100]	0.308	0.346	0.192	0.154	0.000	0.000	0.000	0.000	0.000
(100,150]	0.750	0.000	0.000	0.250	0.000	0.000	0.000	0.000	0.000
(150,200]	0.125	0.000	0.000	0.000	0.625	0.000	0.000	0.250	0.000
(200,250]	0.000	0.000	0.000	0.000	0.000	0.000	0.000	0.000	0.000
(250,350]	0.000	0.000	0.000	0.000	0.000	0.000	0.000	0.000	0.000
(350,500]	1.000	0.000	0.000	0.000	0.000	0.000	0.000	0.000	0.000
(500, +∞)	0.000	0.000	0.000	0.000	0.000	0.000	0.000	0.000	0.000
$p_{\left.s_{i}\right|k}\left(t\right)$	0.805	0.122	0.047	0.007	0.014	0.000	0.000	0.005	0.000
$C_{k}^{s_{i}}\left(t,t+7\right)$	0.008	0.008	0.013	0.000	**0.179**	0.000	0.000	0.000	0.000
$C_{k}\left(t,t+7\right)$	**0.0108**								

f. Complete period ( k=95.188)									
$s_{i}/s_{j}$	[0,25]	(25,50]	(50,100]	(100,150]	(150,200]	(200,250]	(250,350]	(350,500]	(500, +∞)
[0,25]	0.753	0.038	0.046	0.051	0.020	0.018	0.018	0.020	0.037
(25,50]	0.319	0.259	0.203	0.106	0.081	0.000	0.013	0.003	0.016
(50,100]	0.140	0.175	0.337	0.180	0.092	0.037	0.031	0.003	0.005
(100,150]	0.090	0.065	0.285	0.239	0.163	0.061	0.072	0.019	0.006
(150,200]	0.030	0.025	0.219	0.216	0.208	0.134	0.100	0.057	0.010
(200,250]	0.019	0.011	0.094	0.254	0.250	0.151	0.139	0.066	0.016
(250,350]	0.028	0.007	0.049	0.118	0.171	0.191	0.233	0.160	0.044
(350,500]	0.055	0.006	0.020	0.018	0.063	0.129	0.253	0.347	0.108
(500, +∞)	0.172	0.000	0.012	0.025	0.039	0.039	0.088	0.174	0.450
$p_{\left.s_{i}\right|k}\left(t\right)$	0.183	0.040	0.142	0.161	0.140	0.092	0.110	0.081	0.051
$C_{k}^{s_{i}}\left(t,t+7\right)$	0.078	0.058	0.048	0.054	0.062	0.055	0.081	0.100	0.000
$C_{k}\left(t,t+7\right)$	**0.0629**								

It should be noted that upward spatial transition probabilities are grey‐shaded in Table 2, as they are the ones relevant to the estimation of the spatial contagion index. 6 We find that the highest spatial transition probability just before the lockdown (of 0.667) implies that those municipalities that were surrounded by others with high incidence and labelled as ‘low risk’ moved to a situation labelled as ‘high risk’ after 7 days. In any case, this situation was, according to the value of $p_{\left.s_{i}\right|k}\left(t\right)$, merely marginal, since it represented only 1.7% of the municipalities surrounded by others with high incidence. Not surprisingly, during the days prior to the declaration of the lockdown, almost all municipalities (97.1%) started from an idyllic situation of ‘lack of risk’. As is also shown by the row corresponding to $p_{\left.s_{i}\right|k}\left(t\right)$ in Table 2, this situation was reversed during the lockdown, being re‐established, although to a lesser extent, during the following phases. Also noteworthy is the existence of positive upward spatial transition probabilities from most of the intervals towards intervals representing a very high or even extreme risk of contagion during the lockdown (marked in bold), this being a critical condition for the contagion index to reach very high values (even more so if accompanied by high values of $p_{\left.s_{i}\right|k}\left(t\right)$) as evidence of a very rapid spread of the disease in a short period of time.

Concerning the spatial contagion index, we see that its value is very close to zero in all cases. At first glance, this may appear somewhat odd. However, a correct interpretation requires going beyond the theoretical range of values (0–1), since a value of 1 would arise only if ‘all’ transitions were movements towards the most distanced cumulative incidence state, in our case (500, +∞), which is virtually impossible. Thus, for a proper interpretation of the scale of the index, following Hierro et al. (2013) we carried out a number of simulations to distinguish, from a more realistic perspective, between what we consider can be categorized as high, medium and low degrees of spatial contagion. We adopted the following criteria: we labelled a situation as ‘high spatial contagion’ if $C_{k}$ is over 0.0389 (obtained, on average, when 25% of municipalities move to the contiguous state); ‘medium spatial contagion’ if $C_{k}$ is between 0.0389 and 0.0156 (the last threshold obtained when 10% of the municipalities move to the contiguous state); and ‘low spatial contagion’ if $C_{k}$ is below 0.0156. Accordingly, the information provided by Table 2 indicates that spatial contagion reached high values before and during the lockdown, while values were close to the boundary between medium and low contagion throughout the three de‐escalation phases. It should also be noted that, although the intensity of contagion decreased by nearly 20% during the lockdown, the greatest decrease (80%) occurred at the beginning of the de‐escalation phase, stabilizing afterwards until the end of the state of alarm. Consequently, our results show that, despite the long lockdown undergone in Madrid, spatial contagion processes did not decrease in intensity and stabilized until the advent of the de‐escalation phase.

Moving now back to the evidence obtained on spatial dependence, and putting two and two together, our results seem to point to the early spatial contagion processes having triggered a kind of ‘knock‐on effect’ for positive spatial dependence that resulted in a further propagation of the disease across space, while the reverse effect took place once these processes started to fade and became more localized during the de‐escalation phase.

Now, looking at the state‐by‐state indexes, we can determine what type of municipality was more exposed to spatial contagion. As we can see, just before the lockdown, municipalities with no, low or very low COVID‐19 risk were virtually the only ones exposed to contagion processes (values marked in bold in Table 2a). As expected, this picture changed in the lockdown, and spatial contagion spread to all types of municipalities (Table 2b). In contrast, in the de‐escalation phase, transmission processes were more localized, with medium‐risk municipalities being the most exposed (Table 2c–e).

### 4.3 Spread drivers: Conditional spatial contagion index

To complete these results in terms of causality, we next consider the potential influence of some variables linked to community propagation, such as mobility, population and population density, as well as several indicators of social vulnerability, such as gross disposable income and immigration. While the potential influence of the first three variables is straightforward, regarding the latter two it is important to keep in mind that recent studies have highlighted that the unequal distribution of the COVID‐19 outcomes across territories is closely related to economic and social inequalities, with poor and racially segregated places bearing the brunt of the pandemic's burden (Baena‐Díez et al., 2020; Kim & Bostwick, 2020). We speculate that contagion between territories occurs through mobility, but, once it has occurred, the intensity of contagion depends on the characteristics of each territory, captured by population, population density, income and immigration.

As mentioned in the previous section, we computed the new conditional indexes, which must be compared with the previously calculated unconditional ones (Table 3). The Anderson and Goodman (1957) test was applied for each factor f to determine whether the spatial transition probabilities conditioned by factor f are not statistically different from those obtained without its influence. 7 The null hypothesis is mostly rejected at p<0.01, reflecting the importance of these factors, with only two exceptions. First, for the gross disposable income variable, regardless of the period/sub‐period. This result, albeit apparently puzzling, reveals that this type of contagion makes no significant distinction between poor and rich municipalities. Second, for the mobility variable, but restricted to the sub‐periods covering the de‐escalation phases. This result, paradoxically, reinforces the importance of mobility, since it simply reflects that the state of alarm managed to reduce it to a minimum.

**TABLE 3:** Cumulative incidence of COVID‐19 in Madrid: Explanatory analysis based on conditional spatial contagion indexes $C_{\left.k\right|f}\left(t,t+7\right)$

	Before lockdown	Lockdown	De‐escalation: Phase 0	De‐escalation: Phase 1	De‐escalation: Phase 2	Complete period
Mobility conditioned	0.0798^a^	0.0627^a^	0.0114	0.0127	0.0104	0.0886^a^
Population conditioned	0.0874^a^	0.0523^a^	0.0021^a^	0.0055^a^	0.0023^a^	0.0668^a^
Population density conditioned	0.0929^a^	0.0557^a^	0.0013^a^	0.0029^a^	0.0010^a^	0.0733^a^
Income conditioned	0.0717	0.0536	0.0099	0.0117	0.0096	0.0604
Immigration conditioned	0.0881^a^	0.0534^a^	0.0022^a^	0.0044^a^	0.0023^a^	0.0683^a^
**Unconditional index**	**0.0703**	**0.0570**	**0.0115**	**0.0130**	**0.0108**	**0.0629**

*Note*:

^a^ Significant at 1% level.

In addition, the comparison of contagion indexes between factors also reveals that inter‐municipal daily mobility stands out as the main driver of spatial contagion over the whole period, followed at some distance by population density. For sub‐periods, daily mobility plays a major role in the evolution of spatial contagion processes only before and during the lockdown, while it becomes negligible throughout the de‐escalation phase. This confirms that population mobility in the weeks before the lockdown correlates with spatial contagion levels during the lockdown, while its influence declines afterwards.

Finally, our results seem to reveal that the influence of factors such as population, population density and immigration varies greatly over time. While their influence is, to a greater or lesser extent, positive before the lockdown, it becomes negative from then on. This reveals that densely populated municipalities with a large immigrant community stand out as the areas most exposed to spatial contagion processes in the early stage of the pandemic. However, owing to mobility restrictions and the propagation of the pandemic to other areas of the region, this situation reverses over time, with the less densely populated areas and those with smaller immigrant communities becoming the most exposed.

## 5 CONCLUSIONS

The current COVID‐19 pandemic represents an exceptionally complex phenomenon, in which spatial interactions play a major role in its spread through a territory. This paper explored the real dimension, as well as the main driving forces, of the spatial contagion of the COVID‐19 across the municipalities of the region of Madrid, one of the European regions most severely impacted during the first wave of the pandemic. To do so, we proposed a modified spatial Markov chain approach based on the estimation of an unconditional and a conditional spatial contagion index. Accordingly, the two major strengths of the approach are that, on the one hand, it provides a quantitative basis for the rapid detection of high levels of spatial contagion between territories and, on the other hand, it reveals the potential factors contributing to the spatial spread of the disease from high to low incidence areas within a territory.

In this regard, it is worth pointing out that our unconditional contagion indicator revealed the presence of high levels of spatial contagion even before the declaration of the national lockdown, which suggests that the measures were late in coming. Consequently, the computation of a spatial contagion index as the one proposed in the paper could, together with other factors, form part of an early detection system for pandemics, and thus shed light on the need to impose containment measures. Our estimates also confirmed high levels of spatial contagion during the lockdown, which did not diminish and stabilize until well into the de‐escalation phase established by the Spanish government as a bridge to the ‘new normality’. Regarding the main factors behind spatial contagion, the analysis first showed that high inter‐municipal mobility prior to confinement acted as a breeding ground for the presence of positive spatial dependence in subsequent cumulative incidence and, in consequence, for the emergence of major hotspots of COVID‐19. The conditional spatial contagion index also demonstrated that densely populated municipalities with a large immigrant community were the areas most exposed to spatial contagion processes in the early phase of the pandemic in Madrid.

The analysis carried out in this study reveals the need for greater anticipatory power on the part of the authorities when faced with a pandemic, since, as indicated, the measures arrived too late, a conclusion that is valid not only for Madrid but also for many other cases. Moreover, our results call for mobility measures focused on densely populated municipalities with large immigrant communities. Combining the above, a possible strategy, based on frequent counting of these contagion indicators, would consist of imposing mobility restrictions in the most densely populated areas with the highest share of immigrants if the spatial contagion index were to show systematic increases. Should this trend not be curbed, it would be necessary to impose global containment, which, as the paper shows, is effective at controlling pandemics.

## Footnotes

^1^
Data on confirmed COVID‐19 cases for Spain come from the Spanish Ministry of Health (downloaded March 2021).

^2^
We discarded alternative definitions for the spatial weights, such us the contiguity‐based spatial weights, because some municipalities, despite not sharing a border, can exert influence on another (Le Gallo, 2004). We also considered the selection of a cut‐off, defined as the lower quartile of the great distance distribution following Dall'erba et al. (2008), but the results of the Moran's *I* statistic were roughly the same. At any rate, we think that the introduction of a cut‐off is not recommended overall when considering the spatial interactions within a small region, since it may excessively curtail neighbouring influence.

^3^
For a more detailed explanation of a standard spatial Markov chain approach, see Rey (2001).

^4^
Although it is obvious that the average lag in a region takes a different value at each period, so that it depends on t, for the sake of simplicity we shall denote it by k.

^5^
This element controls for differences in relative size between cumulative incidence states. Otherwise, changes in the importance of each state over time would not be included, which would be a major limitation.

^6^
As seen in equation 3, the spatial contagion index is defined for $s_{j}>s_{i}$ because only transitions to intervals of higher cumulative incidence are involved in a process of spatial contagion.

^7^
The corresponding statistic is as follows: ∑si∑sj>sisj∈Fsinsiktpsisjkft,t+z−psisjkt,t+z2psisjkt,t+z, which is asymptotically distributed as $\chi^{2}$ with $\sum_{s_{i}}\left(f_{s_{i}}-1\right)$ degrees of freedom, with $F_{s_{i}}=\left\{s_{j}:p_{\left.s_{i}s_{j}\right|k}\left(t,t+z\right)>0\right\}$, $f_{s_{i}}=\left|F_{s_{i}}\right|$ (i.e., the test is done only for spatial transition probabilities that are positive under the null hypothesis), and $n_{s_{i}|k}\left(t\right)$ being the number of municipalities surrounded by above‐average cumulative incidence ones falling into state $s_{i}$ at t.

## APPENDIX A.

### Some clarifications on the exploitation of data collected from ‘Studies on mobility based on mobile phone’

Despite the richness of data extracted from the ‘Studies on mobility based on mobile phone’ published by the INE, in this study we face two important pitfalls: on the one hand, data are only available for the period 16 March to 20 June; on the other, the level of disaggregation corresponds to what is called mobility areas, which only in some instances matches with municipalities. To have a complete sample, we proceed as follows:

1. For the pre‐lockdown period, we take as reference the data obtained from a pilot study of mobility (INE). Specifically, these data were collected on 18 November 2019.
2. As for the remaining periods, we distinguish the following cases:

- When the mobility areas coincide with the municipality, we directly take the corresponding data.
- For some municipalities having, as mentioned, data at the district level, we simply calculate the mean values between them.
- For those situations in which data are offered for two municipalities jointly, we assign the same data of mobility to both of them.
- The trickiest situation we tackle is when mobility data are given, literally, for ‘a specific municipality (any) and others’. This situation occurs in 14 cases. Here we, first, collect those municipalities without data (that is, under the umbrella of ‘others’ in INE's mobility areas) and, second, assign each to the nearest ‘specific’ municipality.
- Finally, for the case of the municipality of Madrid, although we are already using data at the district level owing to its size, original data are offered here at the census area level. Here, we group all census data that corresponded to each of the 21 districts making up the municipality, calculating mean values between them.
